# Evaluation of high efficiency gene knockout strategies for *Trypanosoma cruzi*

**DOI:** 10.1186/1471-2180-9-90

**Published:** 2009-05-11

**Authors:** Dan Xu, Cecilia Pérez Brandán, Miguel Ángel Basombrío, Rick L Tarleton

**Affiliations:** 1 Department of Cellular Biology and Center for Tropical and Emerging Global Diseases, University of Georgia, Athens, GA 30602, USA; 2 Instituto de Patologia Experimental, Universidad de Salta, 4400 Salta, Argentina

## Abstract

**Background:**

*Trypanosoma cruzi*, a kinetoplastid protozoan parasite that causes Chagas disease, infects approximately 15 million people in Central and South America. In contrast to the substantial *in silico *studies of the *T. cruzi *genome, transcriptome, and proteome, only a few genes have been experimentally characterized and validated, mainly due to the lack of facile methods for gene manipulation needed for reverse genetic studies. Current strategies for gene disruption in *T. cruzi *are tedious and time consuming. In this study we have compared the conventional multi-step cloning technique with two knockout strategies that have been proven to work in other organisms, one-step-PCR- and Multisite Gateway-based systems.

**Results:**

While the one-step-PCR strategy was found to be the fastest method for production of knockout constructs, it does not efficiently target genes of interest using gene-specific sequences of less than 80 nucleotides. Alternatively, the Multisite Gateway based approach is less time-consuming than conventional methods and is able to efficiently and reproducibly delete target genes.

**Conclusion:**

Using the Multisite Gateway strategy, we have rapidly produced constructs that successfully produce specific gene deletions in epimastigotes of *T. cruzi*. This methodology should greatly facilitate reverse genetic studies in *T. cruzi*.

## Background

*Trypanosoma cruzi *is a protozoan parasite and the etiological agent of Chagas disease in humans, also known as American trypanosomiasis. *T. cruzi *infects over 100 species of mammalian hosts and is the leading cause of infection-induced heart failure in Latin America [1,2]. In 2006, approximately 12,500 deaths have been reported as a result of the clinical complications of *T. cruzi*-induced heart disease and the lack of effective treatment [3].

*T. cruzi *has four morphologically and physiologically distinct stages. The bloodstream trypomastigotes and intracellular amastigotes stages of parasites are in the mammalian host, whereas epimastigotes and metacyclic trypomastigotes develop in the insect vector [4]. The diploid genome of *T. cruzi *contains approximately 40 chromosomes encoding a predicted set of 22,570 proteins, of which at least 12,570 represent allelic pairs [5]. Allelic copies of genes in the hybrid CL Brener genome may vary in sequence by as much as 1.5%, and trisomy has also been suggested in the case of some chromosomes [6,7]. Putative functions could be assigned to 50.8% of the predicted protein-coding genes on the basis of significant similarity to previously characterized proteins or known functional domains [5].

In contrast to the substantial *in silico *studies of the *T. cruzi *genome, only 10 genes have been experimentally characterized by reverse genetics in *T. cruzi *[8-18]. These genes were all disrupted through homologous recombination, using a DNA cassette that has a drug selectable marker flanked by the coding sequence or the untranslated regions (UTRs) of the target gene. Although effective, this conventional gene knockout approach not only requires identification of multiple compatible restriction sites for ligation reactions and for vector linearization, it also involves multiple restriction digestions, ligations and cloning steps that make the process cumbersome and time-consuming [19]. Given that RNA interference has, to date, failed to function in *T. cruzi *[20] (in contrast to the situation in the African trypanosomes [21]), a simplified strategy to knockout genes in *T. cruzi *would vastly improve the characterization of the multitude of genes encoding proteins without confirmed or even putative functions.

In this study, we sought to develop a simpler method for the deletion of *T. cruzi *genes. We compared the conventional multi-step knockout technique with two knockout strategies that have been proven to work in other organisms, one-step-PCR- and Multisite Gateway (MS/GW) -based systems. We attempted to knockout the dihydrofolate reductase-thymidylate synthase (*dhfr-ts*) using all three techniques, and enoyl-CoA hydratase (*ech*) genes using the two alternative approaches. Our results show that gene-specific sequences of 78 nucleotides used in one-step-PCR strategy are not sufficient to guarantee homologous recombination in *T. cruzi*. However, the MS/GW-based approach is able to efficiently disrupt target genes. In addition, using the MS/GW strategy, generation of knockout constructs can be completed in as few as 5 days. The results of this study will provide a powerful new tool for reverse genetic studies of *T. cruzi*.

## Results

### *dhfr-ts *gene is disrupted using a conventional KO construct

The *dhfr-ts *gene is annotated as two identical alleles in the diploid CL Brener reference strain and codes for dihydrofolate reductase thymidylate syntase [5]. In most organisms these two enzyme activities are present on separate monofunctional enzymes. In contrast, in *T. cruzi *both enzymes are on the same polypeptide chain, with the DHFR domain at the amino terminus and the TS domain at the carboxy terminus [22,23]. Since these enzymes catalyze consecutive reactions in the *de novo *synthesis of 2'-deoxythymidylate (dTMP), they have been used as targets for chemotherapy, as inhibition of either enzyme disrupts the dTMP cycle and results in thymidine auxotrophy [24-26].

**Figure 1:**
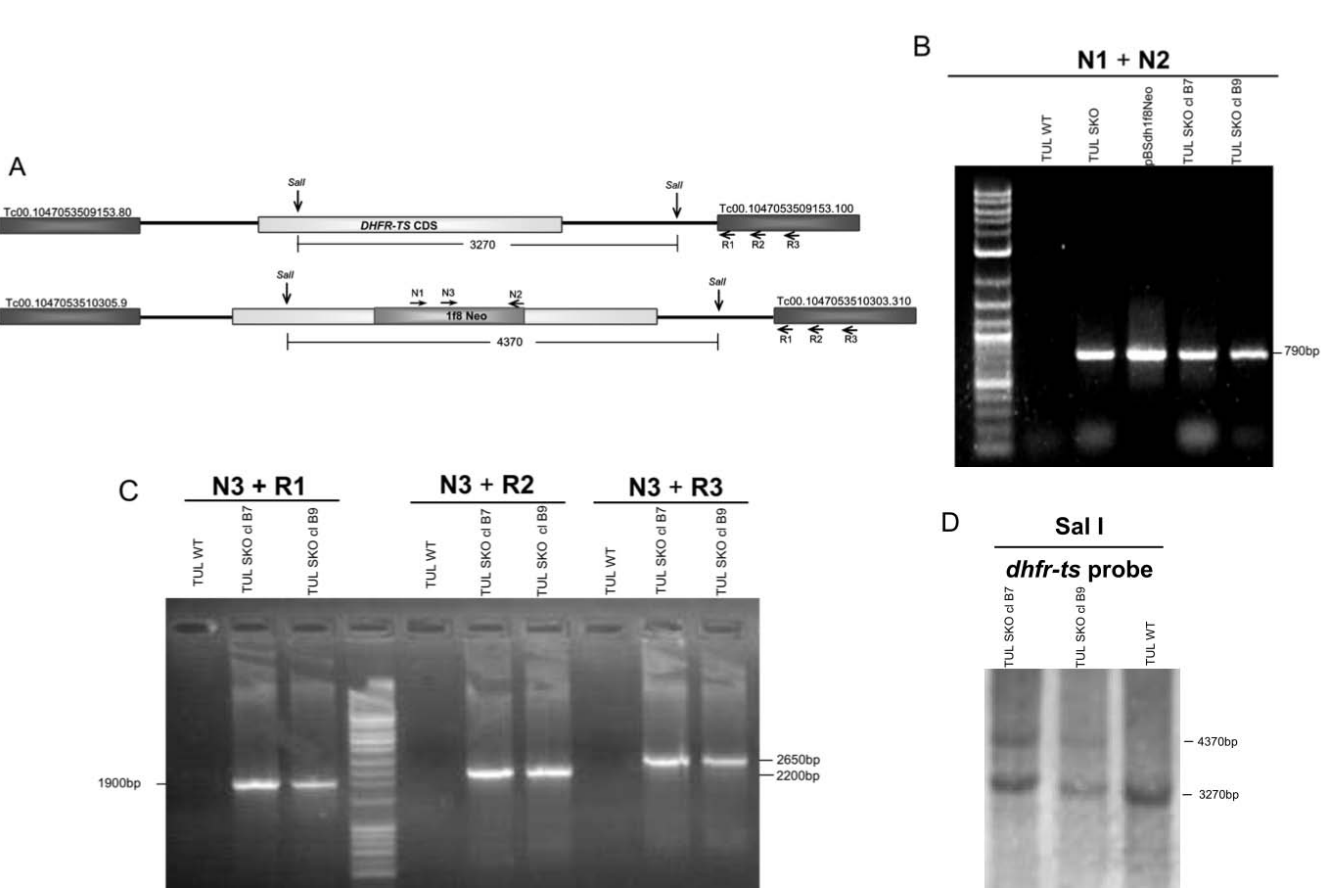
**Disruption of *dhfr-ts *using a conventional KO construct pBSdh1f8Neo**. A) Diagram of the expected genomic loci of *dhfr-ts *and *1f8Neo *in *dhfr-ts*^+/-^/*Neo *parasites. B) PCR analysis with *Neo *specific primers of WT Tulahuen and both uncloned and selected clones of *dhfr-ts*^+/-^/*Neo *parasites. C) PCR analysis with gDNA from selected clones of *dhfr-ts*^+/-^/*Neo *and WT Tulahuen parasites confirming the expected gene disruption of one allele of the *dhfr-ts *gene by *1f8Neo*. D) Southern Blot analysis of WT Tulahuen and two *dhfr-ts*^+/-^/*Neo *clones digested with SalI and probed with *dhfr-ts *probe. Diagram not to scale. Numbers are sizes (bp) of expected products.

G418 (geneticin)-resistant parasites were obtained after transfection of the recombination fragment excised from the plasmid pBSdh1f8Neo (Additional file 1: Figure S1) into the Tulahuen strain of *T. cruzi*. We included a 280 bp 1F8 fraction in the construct so as to provide a trans-splicing acceptor site and a putative polyadenylation signal to the drug resistance gene [27]. Figure 1A shows the expected genomic loci of *dhfr-ts *and *1f8Neo *in *dhfr-ts*^+/-^/*Neo *parasites. As expected no amplification of the *1f8Neo *was observed in Tulahuen WT (wild type) parasites as shown by PCR with primers N1-N2 (Figure 1B). PCR using primers in the flanking genes corroborates the correct insertion of *1f8Neo *gene in *dhfr-ts*^+/- ^parasite's genome. When using N3-R1, N3-R2 and N3-R3 combinations, bands of 1.9, 2.2 and 2.65 kb respectively, were observed, providing further confirmation that the neomycin phosphotransferase gene (*Neo*) had been inserted in the correct locus (Figure 1C). The insertion in the *dhfr-ts *locus was also confirmed by Southern Blot analysis with gDNA from cloned *dhfr-ts*^+/- ^and WT parasites digested with SalI and probed with *dhfr-ts *(Figure 1D). When digested with enzymes SalI and probed with *dhfr-ts *CDS we observe a band of 3.2 kb in wild type parasites while mutants have a 1092 bp insertion corresponding to the *1f8Neo *cassette interrupting the *dhfr-ts *CDS, resulting in an extra 4.4 kb band in the mutants.

### *dhfr-ts *gene is replaced using a MS/GW construct

Since we were able to obtain *dhfr-ts*^+/- ^parasites we concluded that this gene would be a good candidate to evaluate the one-step-PCR and Multisite Gateway-based systems for gene knockout constructs in *T. cruzi*. In the MS/GW recombination fragments, the flanking regions of the gene were used as arms for recombination event, in contrast with the method in Figure 1 where the coding sequence of the gene was used for homologous recombination.

**Figure 2:**
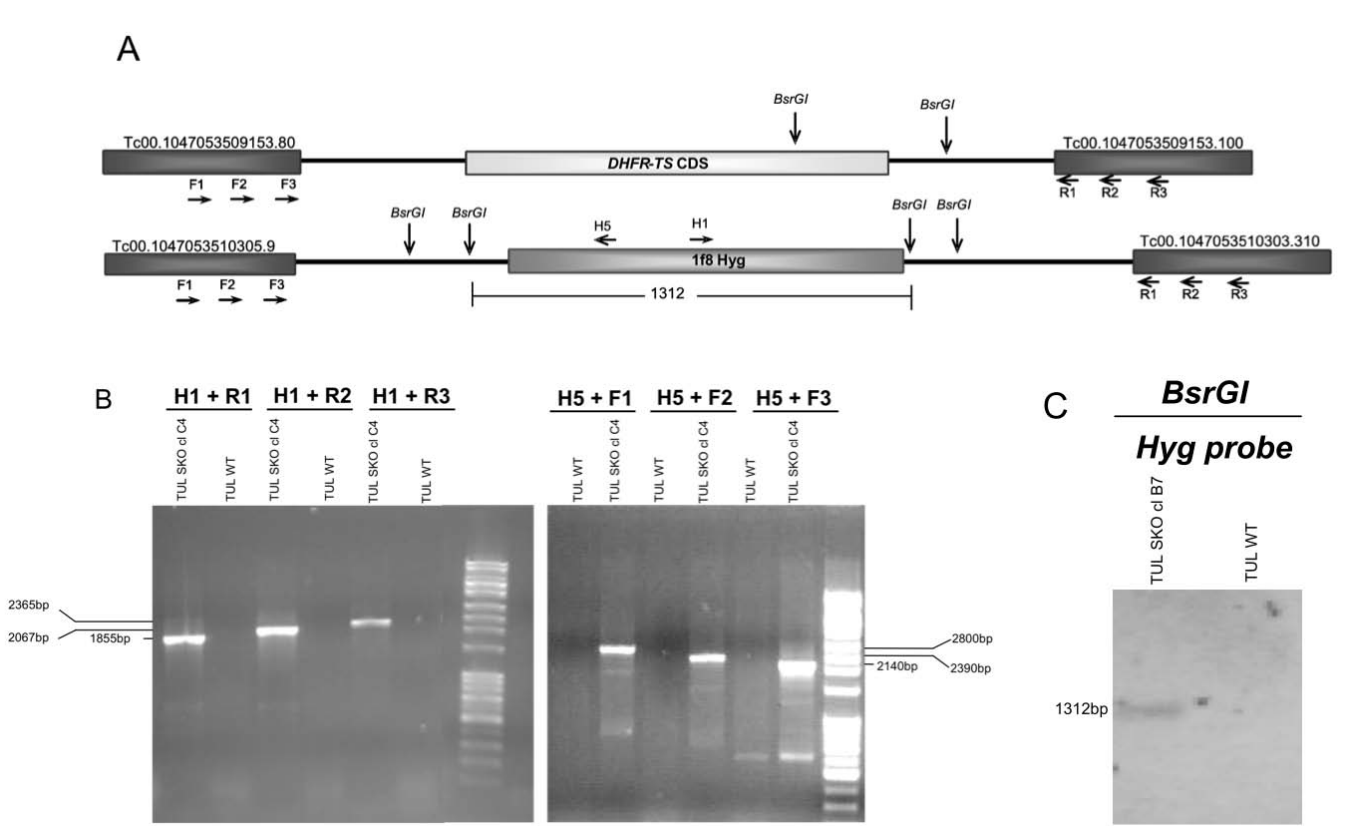
**Replacement of dhfr-ts gene with a MS/GW construct pDEST/dhfr-ts_1F8Hyg**. A) Schematic of the expected genomic loci of *dhfr-ts *and *1f8Hyg *in *dhfr-ts*^+/-^/*Hyg *parasites. B) PCR analysis with gDNA from cloned drug resistant parasites and WT Tulahuen parasites confirm the expected gene deletion of one allele of the *dhfr-ts *gene and correct insertion of *1f8Hyg*. Primer H1 plus the R1, R2 or R3 downstream primers, yield the expected products of 1.8, 2.0 and 2.3 kb, respectively and the combination of H5 plus upstream primers F3, F2 and F1 give the predicted bands of 2.1, 2.4 and 2.8 kb for respectively. See additional file 3: Table S5 for nucleotide sequences of primers. C) Genomic DNA Southern blot analysis of a *dhfr-ts*^+/-^/*Hyg *Tulahuen clone. gDNA digested with BsrGI and hybridized with labeled Hyg CDS probe. Diagram not to scale. Numbers are sizes (bp) of expected products.

Drug resistant lines produced by the transfection of Tulahuen strain epimastigotes with a recombination fragment obtained from pDEST/dhfr-ts_1F8Hyg plasmid (Additional file 2: Figure S2) were cloned and analyzed by PCR and Southern Blot. Figure 2A shows the expected genomic loci of *dhfr-ts *and *1f8Hyg *in the genome of *dhfr-ts*^+/-^/*Hyg *parasites; the results of PCR analysis (Figure 2B) confirm the correct insertion of *1f8Hyg *replacing one allele of the *dhfr-ts *gene (Additional file 3). Southern Blot analysis also showed correct insertion of the *1f8Hyg *cassette replacing one copy of the *dhfr-ts *gene in the genome. The expected 1312 bp band was observed in BsrGI digested DNA from *dhfr-ts*^+/- ^cloned parasites and probed with *Hyg *(hygromycin resistance gene) CDS but not in the WT parasites (Figure 2C).

### Consecutive *ech1 *and *ech2 *genes are simultaneously replaced by constructs generated based on MS/GW system

**Figure 3:**
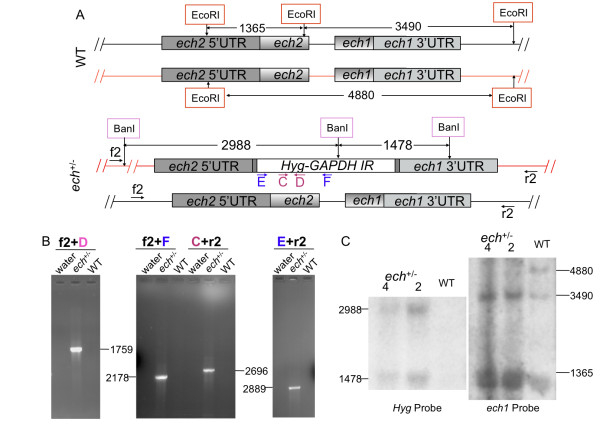
**Simultaneous replacement of consecutive *ech1 *and *ech2 *genes by a MS/GW construct pDEST/*ech*-Hyg-GAPDH**. A) Diagram of *ech1*, *ech2 *and *Hyg-GAPDH-IR *genomic loci in WT and *ech*^+/-^/*Hyg *parasites. B) PCR genotyping analysis of: no template control (water); *ech*^+/-^/*Hyg *(*ech*^+/-^) and WT CL (WT). See additional file 3: Table S5 for nucleotide sequences of primers. C) Southern blot analysis of two clones (2 and 4) of *ech*^+/-^/*Hyg*. Left panel, gDNA digested with BanI and hybridized with *Hyg *CDS; right panel, gDNA digested with EcoRI and hybridized with labeled *ech1 *CDS. Diagrams not to scale. Numbers are sizes (bp) of expected products.

*T. cruzi ech1 *and *ech2 *are tandemly arranged genes (Figure 3A) with a nucleotide sequence identity of 67%. Both genes encode putative enoyl-CoA hydratase/isomerase (ECH) family proteins, which catalyze the second step in the beta-oxidation pathway of fatty acid metabolism. Analysis of the *T. cruzi *proteome suggested that enzymes in the fatty acid oxidation pathway, including ECH, are preferentially expressed in amastigotes [28]. Therefore, we hypothesized that we would be able to knockout both *ech1 *and *ech2 *genes in epimastigotes. The *ech *locus also provides an opportunity to test whether or not the MS/GW approach can be used to produce knockouts of multiple genes that are physically linked in the genome.

In *T. cruzi*, transcript stability and protein translation is largely controlled by 3'UTR and intergenic regions [29,30]. The intergenic region of a constitutively expressed gene, *gapdh*, gives consistently high levels of stable RNA in different constructs and in different life cycle stages [31]. Hence, we included the 3' UTR of *gapdh *in our constructs, to ensure the expression of the inserted drug resistant genes in the epimastigote stage. Transfection of the DNA fragment from pDEST/*ech*-Hyg-GAPDH (Additional file 4: Figure S3A) resulted in parasite lines that were resistant to Hyg selection. Figure 3A shows the expected genomic loci of *ech *and *Hyg-GAPDH-IR *in the genome of *ech*^+/-^/*Hyg *parasites. PCR analysis with the genomic DNA from the drug resistant parasites and WT CL confirmed the expected gene replacement of *ech1 *and *ech2 *genes by *Hyg-GAPDH-IR *(Figure 3B); no products were obtained when using WT CL gDNA as the template with primer combinations f2 and D, f2 and F, C and r2, and E and r2, whereas products of the expected sizes, 1759 bp, 2178 bp, 2696 bp and 2889 bp, respectively, were observed with gDNA from *ech*^+/-^/*Hyg *as the template. Southern blot analysis of EcoR I digested gDNA using the *ech1 *gene as a probe (Figure 3A and 3C right panel) showed a 4880 bp band corresponding to the replaced allelic copy of both *ech *genes was undetected in *ech*^+/-^/*Hyg*, whereas the 3490 bp and 1365 bp bands corresponding to the second allele were retained. In addition, a 2988 bp band and a 1478 bp band corresponding to the inserted *Hyg-GAPDH-IR *were observed in BanI digested gDNA of only the *ech*^+/-^/*Hyg*, but not that of WT CL (Figure 3A and 3C left panel). Taken together, these results confirmed that one copy of each of the tandem *ech1 *and *ech2 *genes was replaced by the MS/GW *Hyg-GAPDH-IR *knockout cassette.

**Figure 4:**
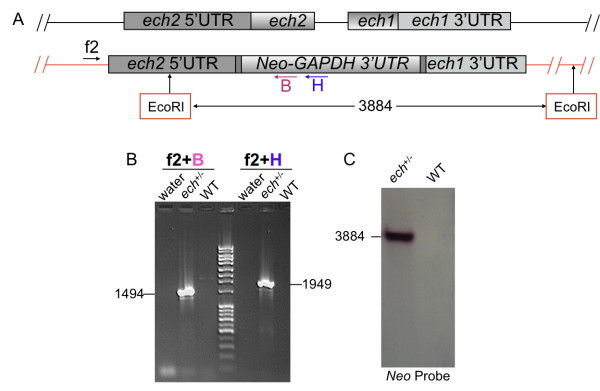
**Simultaneous replacement of consecutive *ech1 *and *ech2 *genes by another MS/GW construct pDEST/*ech*_Neo-GAPDH**. A) Diagram of *ech1*, *ech2 *and *Neo-GAPDH 3'UTR *genomic loci in *ech*^+/-^/*Neo *parasites. B) PCR genotyping analysis of: no template control (water); *ech*^+/-^/*Neo *(*ech*^+/-^) and WT CL (WT). See additional file 3: Table S5 for nucleotide sequences of primers. C) Southern blot analysis of WT CL (WT) and *ech*^+/-^/*Neo *(*ech*^+/-^) digested with EcoRI and hybridized with *Neo *CDS. Diagram not to scale. Numbers are sizes (bp) of expected products.

Similarly, using linearized DNA from pDEST/*ech*_Neo-GAPDH (Additional file 4: Figure S3B), we generated *ech*^+/-^/*Neo *parasites with one copy of both *ech1 *and *ech2 *gene replaced by *Neo-GAPDH-3'UTR *knockout cassette (Figure 4A). This result is confirmed by both PCR amplification (Figure 4B) of gDNA of the drug resistant parasites, as PCR with primer combinations f2 and B, and f2 and H generated 1494 bp and 1949 bp bands respectively only in drug resistant parasites. Southern blot hybridization also showed a 3884 bp *Neo *gene band in the *ech*^+/-^/*Neo *parasites (Figure 4C).

### One-step-PCR knockout strategy fails to delete *dhfr-ts *and ech genes

Since we demonstrated that at least one allele of the *dhfr-ts *can be deleted using the MS/GW based system, we next tested if this gene can be deleted using the one-step-PCR strategy. Transfection and selection of parasites with the knockout cassette LP-*dhfr-ts*-Neo failed to yield drug resistant parasites, despite 4 independent attempts. As there are 78 nts of the CDS of *dhfr-ts *gene in both forward long primers used to produce LP-*dhfr-ts*-Neo, the drug selectable markers were to be expressed as a fusion protein, with 26 amino acids of the start of *dhfr-ts *gene fused at the N terminal. It is possible that the knockout parasites were not obtained because the drug selectable marker has reduced enzyme activity when expressed as a fusion protein. To exclude this possibility, we constructed LP-*dhfr-ts*-UTR-Neo to completely delete the entire *dhfr-ts *sequence. This construct has 78 nts of the UTR of *dhfr-ts *gene instead of the CDS, providing production of neomycin phosphotransferase as a non-fusion protein. However, as with the previous construction, no resistant parasites could be obtained despite 4 independent electroporations. Furthermore, one-step-PCR strategy also failed to delete the *ech1 *and *ech2 *genes despite 5 independent transfection and selection attempts. Therefore, the constructs generated with one-step-PCR strategy that bear 78 nts gene CDS or UTR specific sequence are likely to be insufficient for homologous recombination in *T. cruzi*.

## Discussion

Experimental characterization of gene functions in trypanosomatids has relied heavily on reverse genetic approaches and has been facilitated by the development and optimization of gene manipulation strategies and transfection protocols [30]. In contrast to the robust and extensive techniques for genetic manipulation documented in *Trypanosoma brucei *and *Leishmania*, the validated techniques and record of success for *T. cruzi *is much less extensive. A goal of this study was to validate gene KO strategies for *T. cruzi *which might facilitate research on this important cause of human disease.

Toward that end, we have compared a conventional multi-step cloning technique with two knockout strategies that have been proven to target gene deletion in other organisms, one-step-PCR and MultiSite Gateway. The appeal of the one-step-PCR- strategy is the speed with which constructs can be produced. However, the attempts to knockout either *ech *or *dhfr-ts *genes in *T. cruzi *using this approach were unsuccessful, presumably because the 78 nucleotide-gene-specific regions used in our constructs were insufficient for homologous recombination in *T. cruzi*. This result is perhaps not surprising as studies in *Leishmania *[32] demonstrated that at least 150 nucleotides are needed to guide homologous recombination. However, a recombination rate of 4 × 10^-4 ^was obtained with as short as 42 nucleotides homology in *T. brucei *[33]. Because of the considerable expense of oligos of >100 bp, we did not investigate the minimum length needed for consistent recombination in *T. cruzi*, believing such an approach to be impractical for economical, high-efficiency gene knockouts.

**Figure 5:**
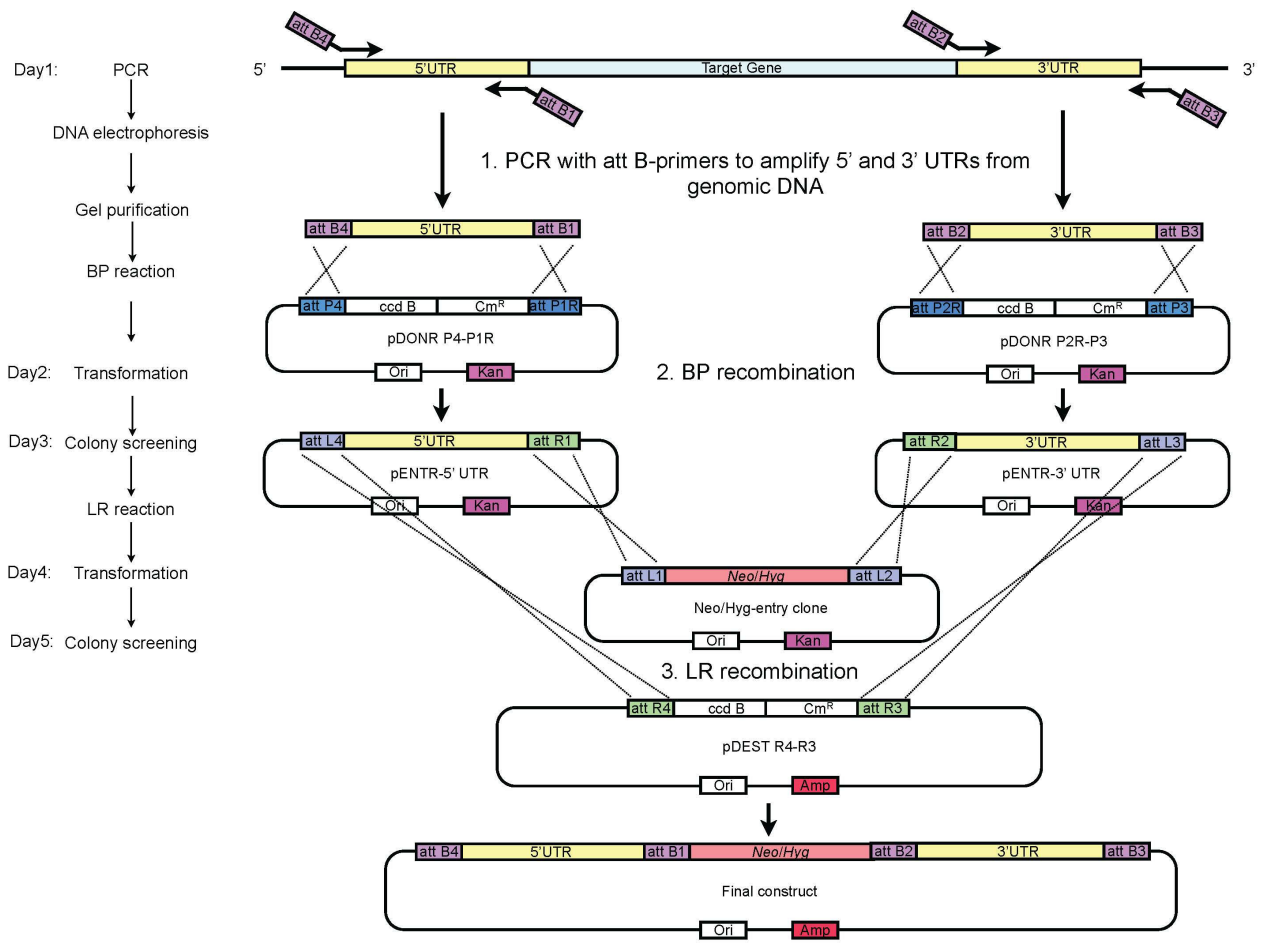
**Timeline for constructing a KO plasmids using MS/GW strategy**. The Multisite Gateway based method consists of three steps: 1) PCR with attB-containing primers to amplify 5' and 3' UTR from genomic DNA; 2) BP recombination of each PCR products with specific donor vectors to generate entry clones containing the UTRs; 3) LR recombination of the two entry clones made in step 2 and a third entry clone containing Neo/Hyg to create the final construct. (Kan, kanamycin-resistance gene; Amp, ampicillin-resistance gene; Ori, Origin of replication).

The MultiSite Gateway-based approach, although not as simple as the one-step-PCR strategy, is far less time-consuming than the standard conventional methods. In particular, extensive restriction mapping, digestion and ligation steps are not needed at all with the MS/GW approach [34]. pDONR vectors containing drug resistance genes can be generated once and then repeatedly re-used for production of knockout constructs for different genes, further increasing the efficiency of the process. Once regions flanking the genes of interest are obtained from the *att*- PCR amplifications, the knockout DNA constructs can be generated within as few as five days (Figure 5). The BP and LR reactions are robust and have very high success rates; typically, at least 90% colonies screened from our BP and LR reactions are positive. Using the MS/GW knockout constructs, we successfully obtained *dhfr-ts*^+/- ^and *ech*^+/- ^parasites in two different *T. cruzi *strains. In on-going work, we have used MS/GW constructs to successfully produce single as well as double KO lines for more than 10 other genes, ranging in size from 828 to 2730 nucleotides and up to 3 copies (using additional drug resistance markers). Thus the MS/GW approach appears to be amenable to use as part of a higher throughput gene knockout project.

Overall, the results described here identify the Multisite Gateway (MS/GW) -based system as an efficient tool to create knockout construction for deletion of genes in *T. cruzi *and should help accelerate the functional analysis of a wider array of genes in this important agent of disease.

## Conclusion

This study documents the development of a Multisite Gateway based method for efficient gene knockout in *T. cruzi*. Further, we demonstrate that long-primer-based KO constructs with <80 nucleotides of homologous gene sequences are insufficient for consistent homologous recombination in *T. cruzi*. The increase in efficiency of gene knockout constructs should facilitate increased throughput for the identification of gene function in *T. cruzi *using reverse genetics.

## Methods

### Culture, transfection and cloning of *T. cruzi*

CL and Tulahuen lines of *T. cruzi *epimastigotes were cultured at 26°C in supplemented liver digest-neutralized tryptose (LDNT) medium as described previously [35]. A total of 1 × 10^7 ^early-log epimastigotes were centrifuged at 1,620 g for 15 min and resuspended in 100 μl room temperature Human T Cell Nucleofector™ Solution (Amaxa AG, Cologne, Germany). The resuspended parasites were then mixed with 3–10 μg DNA (8–10 μg DNA for constructs generated using the conventional and MS/GW strategy; 3–10 μg DNA for constructs generated through one-step-PCR) in a total volume of 5–10 μl and electroporated using program "U-33" in an AMAXA Nucleofector Device. This protocol generally yields 6–13% yellow fluorescent protein (YFP) positive parasites 24 hrs after transfection using 10 μg of a YFP-containing control plasmid. The electroporated parasites are cultured in 25 cm^2 ^cell culture flasks (Corning Incorporated, Lowell, MA, USA) with 10 ml LDNT medium; 250 μg/ml G418 (for transfectants with neomycin phosphotransferase gene-containing cassette) and/or 600 μg/ml Hyg (Hygromycin B, for transfectants with hygromycin reisitance gene-containing cassette) was added at 24 hrs post-transfection. Parasites were considered fully selected when parasites transfected with no DNA were dead, generally at 4–5 weeks post-transfection. For single-cell cloning, drug selected lines were deposited into a 96-well plate to a density of 1 cell/well using a MoFlow (Dako-Cytomation, Denmark) cell sorter and cultured in 250 μl LDNT supplemented with G418 or Hyg. Each population from an individual well was considered an individual clone.

### Construction of a knockout DNA cassette using the conventional strategy

The complete coding sequence of 1566 bp of the *dhfr-ts *gene was amplified by PCR from genomic DNA (gDNA) of the WT Tulahuen strain using AmpliTaq Gold^® ^DNA Polymerase (Roche) and primers DH5_f and DH6_r (Additional file 5: Table S1). The PCR product was gel purified with QIAquick Gel Extraction Kit (Qiagen, Valencia, CA, USA), treated with T4 DNA Polymerase (BioLabs) to generate blunt ends and cloned into the KpnI-digested, T4 DNA Polymerase (BioLabs) treated and dephosphorylated pBlueScript SKII + (Stratagene). Then the *dhfr-ts *coding region was disrupted by inserting into the PshAI restriction site of the *dhfr-ts *gene the neomycin phosphotransferase gene which have been generated by digestion with NotI/StuI of pBSSK-neo1f8 plasmid [27]. The resulting recombination vector were sequenced and designated as pBSdh1f8Neo (Additional file 1: Figure S1) containing the *Neo *CDS plus the trans-splicing 1f8 region, as well as 1016 bp and 550 bp of the 5' and 3'*dhfr-ts *coding regions.

The final plasmid was digested with restriction enzyme KpnI to liberate the knockout DNA cassette, gel eluted, ethanol precipitated and resuspended in water to a final concentration of 1–2 μg/μl.

### Construction of knockout DNA cassettes based on MS/GW strategy

All plasmids were constructed based on MS/GW system using commercially available MultiSite Gateway Three-Fragment Vector Construction kit (Invitrogen, Carlsbad, CA, USA), which includes all the Entry vectors and Destination vectors used in this study (Figure 5). In the Gateway system, the "BP" reaction is a recombination reaction between attB and attP sites in the PCR fragment and Donor vectors, resulting in Entry clones contains the gene of interest flanked by attL sites. "LR" reactions allow recombination between attL and attR sites of a Destination vector to yield an expression clone.

### pDEST/dhfr-ts_1F8Hyg

In order to construct the pDEST/dhfr-ts_1F8Hyg plasmid, 1.0-kb 5' flanking sequence of *dhfr-ts *was amplified from gDNA of the WT CL strain using primers attB4_5'UTR_dhfr_f and attB1_5'UTR_dhfr_r (Additional file 6: Table S2) and Platinum^® ^PCR SuperMix (Invitrogen), gel purified with QIAquick Gel Extraction Kit (Qiagen, Valencia, CA, USA) and cloned into the Entry vector pDONR™P4-P1R through a BP reaction using the BP Clonase II Enzyme Mix (Invitrogen), resulting in the Entry clone pDONR_5'UTR_dhfr. Similarly, 1.0-kb 3' flanking sequence of *dhfr-ts *was amplified using primers attB2_3'UTR_dhfr_f and attB3_3'UTR_dhfr_r (Additional file 6: Table S2) and cloned into pDONR™P2R-P3 to generate pDONR_3'UTR_dhfr. Using plasmid pBSSK-hyg1f8 [27] as a template, the *Hyg *and its upstream 1f8 region was amplified with primers attB1_1F8_f and attB2_1F8Hyg_r (Additional file 6: Table S2) and cloned into Entry vector pDONR™221. The three Entry clones were then mixed with a Destination vector pDEST™R4-R3 in an LR reaction using the LR Clonase II Plus Enzyme Mix (Invitrogen) to generate a final plasmid pDEST/dhfr-ts_1F8Hyg (Additional file 2: Figure S2). The knockout DNA cassette was liberated from the plasmid backbone with AlwNI and PvuI enzymes, and purified as above.

### pDEST/*ech*_Neo-GAPDH and pDEST/*ech*_Hyg-GAPDH

*Trypanosoma cruzi ech1 *and *ech2 *are tandemly arranged genes. To construct the pDEST/*ech*_Hyg-GAPDH plasmid, 1.0-kb 5' sequence of *ech2 *was amplified with primers attB4_ech5'UTR_f and attB1_ech5'UTR_r (Additional file 6: Table S2), gel purified and cloned into the Entry clone pDONR-ech5'UTR. Similarly, 1.0-kb 3' sequence of *ech1 *was amplified with primers attB2_ech3'UTR_f and attB3_ech3'UTR_r (Additional file 6: Table S2) and cloned into pDONR™P2R-P3 to generate pDONR-ech3'UTR. *Hyg *and the downstream intergenic region of *GAPDH *(glyceraldehyde-3-phosphate dehydrogenase) (*GAPDH-IR*) was amplified from plasmid pTEX-Hyg.mcs [36] using primers attB1_Hyg_f and attB2_Hyg_r (Additional file 6: Table S2) and cloned into Entry vector pDONR™221. The three Entry clones were then mixed with a Destination vector pDEST™R4-R3 to generate pDEST/*ech*_Hyg-GAPDH (Additional file 4: Figure S3A) through a LR reaction. The final plasmid was digested with restriction enzymes PvuII and PciI and purified as above.

Similarly, to construct pDEST/*ech*_Neo-GAPDH (Additional file 4: Figure S3B), *Neo *and 3'UTR of *GAPDH *(*GAPDH *3'UTR) was amplified from plasmid pTrex-YFP (modified from the backbone of pTrex [37]) with primers attB1_Neo_f and attB2_Neo_r (Additional file 6: Table S2) and cloned into Entry vector pDONR™221. The final plasmid was digested with restriction enzymes PvuI and PciI and purified as above.

### Construction of knockout DNA cassettes via one-step-PCR

For the constructs for deletion of the *dhfr-ts *gene using one-step-PCR, *Neo *and *Hyg *was amplified with primers LP_dhfr_Neo_f and LP_dhfr_Neo_r, and LP_dhfr_Hyg_f and LP_dhfr_Hyg_r (Additional file 7: Table S3) from plasmids pTrex-YFP and pTEX-Hyg.mcs respectively. In both cases, forward primers and reverse primers corresponded to the 78 bp downstream of the start codon of the *dhfr-ts *gene and reverse 78 bp upstream of the stop codon of the gene, respectively.

In addition, primers LP_dhfr-UTR_Neo_f and LP_dhfr-UTR_Neo_r, (Additional file 7: Table S3) were also used to amplify *Neo *from pTrex-YFP. In this case, LP_dhfr-UTR_Neo_f included 78 bp upstream of the start codon of the *dhfr-ts *gene whereas LP_dhfr-UTR_Neo_r bears 78 bp downstream of the stop codon.

Likewise, primers LP_ech_Neo_f and LP_ech_Neo_r (Additional file 7: Table S3) were designed to amplify the final construction for deletion of the *ech *genes as well as primers LP_ech_Hyg_f and LP_ech_Hyg_r (Additional file 7: Table S3). PCR reactions were carried out as follows: initial denaturation at 94°C for 3 min followed by 30 cycles of: 98°C for 20s; 55°C for 30s; and 72°C for 2 min followed by 72°C for 10 min using Gradient Master Thermocycler (Eppendorf, Westbury, NY, USA). Products were collected and purified with QIAquick PCR Purification Kit. The eluted DNA was further ethanol precipitated and resuspended to 0.2–1 μg/μl.

### Southern blot

For Southern blot analysis, gDNA from different clones and strains was purified using Wizard Genomic DNA Purification Kit (Promega Corporation, Madison, WI, USA). The DNA was digested and separated by 0.7% agarose gel electrophoresis, and the gels blotted onto nylon membranes (Hybond-N 0.45-mm-pore-size filters; Amersham Life Science) using standard methods [38]. For probe generation, a 1030 bp DNA (*Hyg*) was amplified using primers Hyg_f and Hyg_r (Additional file 8: Table S4) from plasmid pTEX-Hyg.mcs. For the *Neo *probe, a 795 bp DNA fragment was amplified from plasmid pBSSK-neo1f8 using primers Neo_f and Neo_r (Additional file 8: Table S4). *ech1 *gene were amplified using primers ech1_pb_f and ech1_pb_r (Additional file 8: Table S4) from gDNA of WT CL, while *dhfr-ts *gene was amplified from gDNA of WT Tulahuen using primers DH5_f and DH6_r (Additional file 5: Table S1). The PCR products were purified as above. Labeling of the probe and DNA hybridization were performed according to the protocol supplied with the PCR-DIG DNA-labeling and detection kit (Roche Applied Science, Indianapolis, IN, USA).

## Authors' contributions

DX participated in the design of the study, carried out the *ech *gene knockout experiments, and drafted the manuscript. CPB participated in the design of the study, carried out the experiments to knockout the *dhfr-ts *gene, and revised this manuscript intensively. MAB participated in its design and coordination and revised the manuscript critically. RLT conceived of the study, participated in its design and coordination and revised the manuscript critically. All authors read and approved the final manuscript.

## Supplementary Material

Additional File 1 **Figure S1**. Plasmid map of pBSdh1f8Neo for conventional disruption of the *dhfr-ts *gene. Click here for file

Additional File 2 **Figure S2**. Plasmid map of pDEST/dhfr-ts_1F8Hyg obtained by the MS/GW system used for the deletion of the *dhfr-ts *gene. Click here for file

Additional File 3 **Table S5**. Oligonucleotides for PCR analysis. Click here for file

Additional File 4 **Figure S3**. Maps of the plasmids obtained by the MS/GW system used for the deletion of the *ech *gene. A) pDEST/*ech*_Hyg-GAPDH and B) pDEST/*ech*_Neo-GAPDH. Click here for file

Additional File 5 **Table S1**. Oligonucleotides for generation of knockout constructs based on the conventional strategy. Click here for file

Additional File 6 **Table S2**. Oligonucleotides for generation of knockout constructs based on the MS/GW strategy. Click here for file

Additional File 7 **Table S3**. Oligonucleotides for one-step-PCR. Click here for file

Additional File 8 **Table S4**. Oligonucleotides for probe generation of Southern blot analysis. Click here for file
