# Supplementary figures and images for: Evaluation of high efficiency gene knockout strategies for Trypanosoma cruzi
Source: BMC Microbiol. 2009 May 11;9:90. doi: 10.1186/1471-2180-9-90 (PMC2688506; doi:10.1186/1471-2180-9-90)

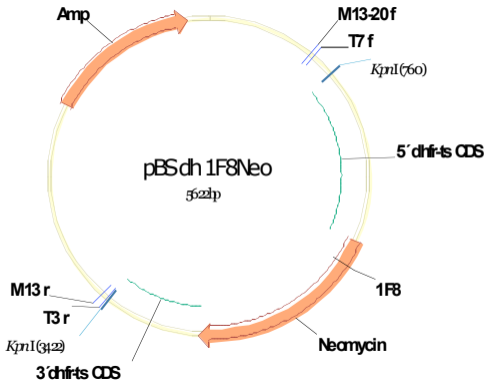

Supplement: Additional File 1 — Figure S1. Plasmid map of pBSdh1f8Neo for conventional disruption of the dhfr-ts gene. [file 1471-2180-9-90-S1.pdf]

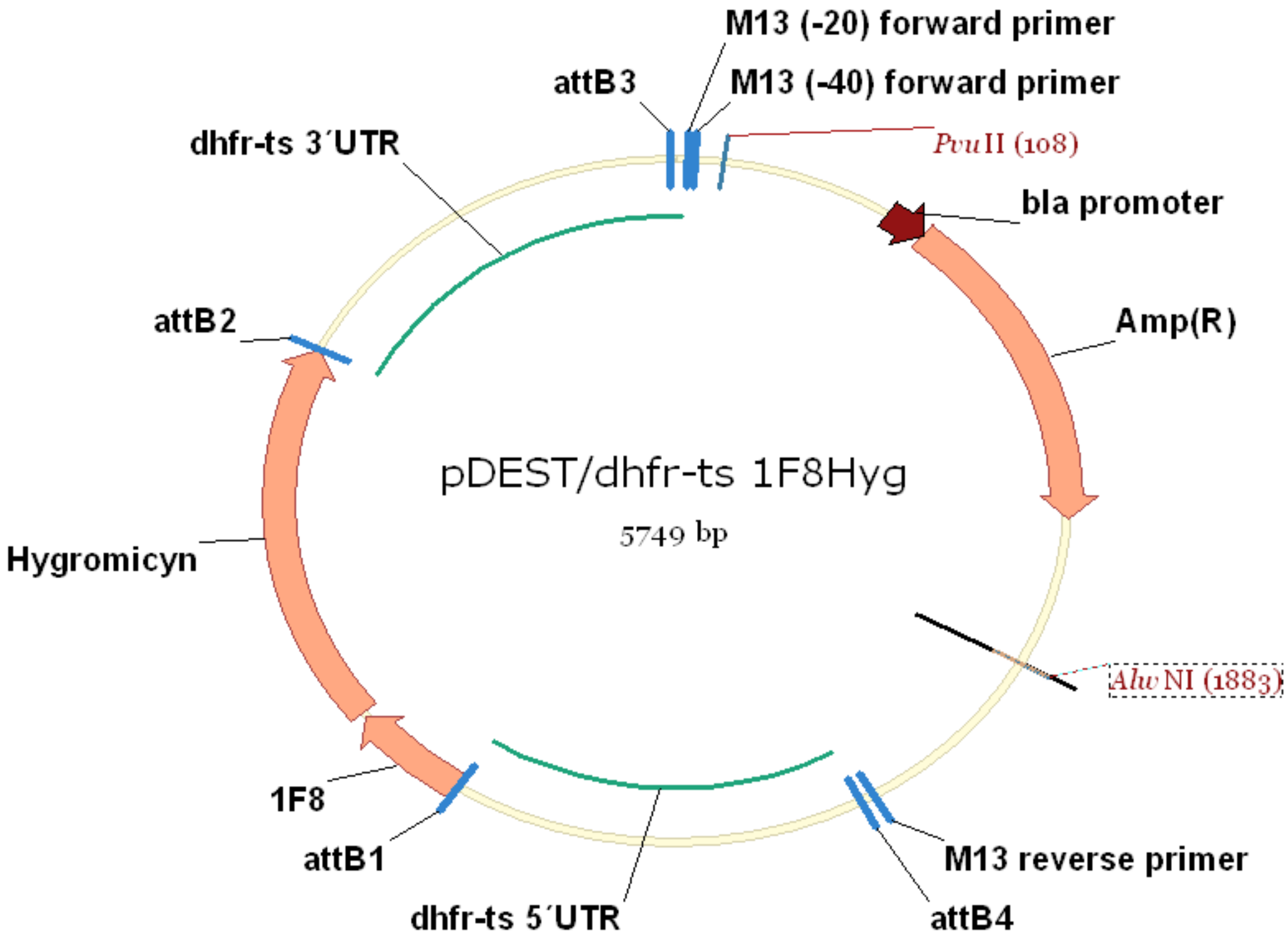

Supplement: Additional File 2 — Figure S2. Plasmid map of pDEST/dhfr-ts_1F8Hyg obtained by the MS/GW system used for the deletion of the dhfr-ts gene. [file 1471-2180-9-90-S2.pdf]

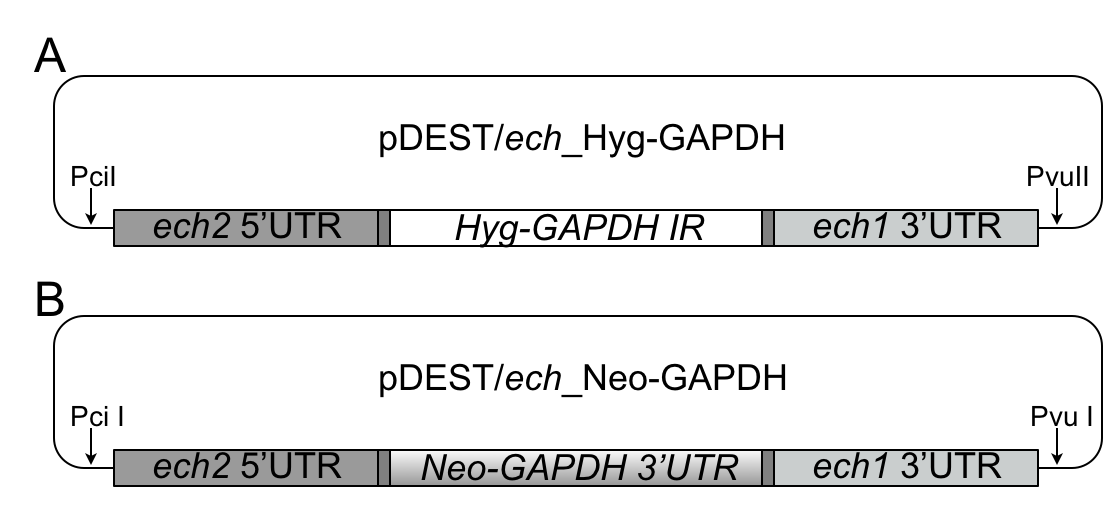

Supplement: Additional File 4 — Figure S3. Maps of the plasmids obtained by the MS/GW system used for the deletion of the ech gene. A) pDEST/ech_Hyg-GAPDH and B) pDEST/ech_Neo-GAPDH. [file 1471-2180-9-90-S4.tiff]
