# Supplementary material for: Evaluation of high efficiency gene knockout strategies for Trypanosoma cruzi
Source: BMC Microbiol. 2009 May 11;9:90. doi: 10.1186/1471-2180-9-90 (PMC2688506; doi:10.1186/1471-2180-9-90)
Supplement: Additional File 3 — Table S5. Oligonucleotides for PCR analysis. [file 1471-2180-9-90-S3.doc]

Supplementary table 5. Oligonucleotides for PCR analysis

| Name | Sequence |
| --- | --- |
| B | ATACTTTCTCGGCAGGAGCA |
| C | CGCAAGGAATCGGTCAATAC |
| D | ACATTGTTGGAGCCGAAATC |
| E | ATGAAAAAGCCTGAACTCACC |
| F | TACTCTATTCCTTTGCCCTC |
| H | TCAGAAGAACTCGTCAAGAAG |
| f2 | CACATGCGTTATTATGGTCC |
| r2 | TATTACCCGAGACATTGACG |
| R1  R2  R3  F1  F2  F3 | CGACTTTCACTTTGCCATGA  ATGGTGTCGGAGATTCTTGG  ATTGTGTTTGCGAAGCAGT  AAGGGTGATGCCGTGATAAG  GATCCAACCAACTGGAGGAA  CTTCGAGGAGCTTTGCTGTT |
| N1 | ATGATTGAAC AAGATGGATT |
| N2 | agaactcgtcaagaaggcga |
| N3 | ATCTGGACGAA GAGCATCAGG |
| H1 | CGTC TGTCGAGAAG TTTCTG |
| H5 | ACAGCGTCTCC GACCTGAT |
