# Supplementary material for: Evaluation of high efficiency gene knockout strategies for Trypanosoma cruzi
Source: BMC Microbiol. 2009 May 11;9:90. doi: 10.1186/1471-2180-9-90 (PMC2688506; doi:10.1186/1471-2180-9-90)
Supplement: Additional File 5 — Table S1. Oligonucleotides for generation of knockout constructs based on the conventional strategy. [file 1471-2180-9-90-S5.doc]

Supplementary table 1. Oligonucleotides for generation of knockout constructs based on the conventional strategy.

| Name | Sequence |
| --- | --- |
| DH5_f | TGTCGCTGTTTAAGATCCGC |
| DH6_r | CCATGAAGATGGCGGTTTAG |
