# Supplementary material for: Evaluation of high efficiency gene knockout strategies for Trypanosoma cruzi
Source: BMC Microbiol. 2009 May 11;9:90. doi: 10.1186/1471-2180-9-90 (PMC2688506; doi:10.1186/1471-2180-9-90)
Supplement: Additional File 6 — Table S2. Oligonucleotides for generation of knockout constructs based on the MS/GW strategy. [file 1471-2180-9-90-S6.doc]

Supplementary table 2. Oligonucleotides for generation of knockout constructs based on the MS/GW strategy.

| Name  attB4_5´UTR_dhfr_f  attB1_5´UTR_dhfr_r  attB2_3´UTR_dhfr_f | Sequence  ggggacaactttgtatagaaaagttgtgcacactcacaccaatactca  ggggactgcttttttgtacaaacttgggtggacgcacaagtgagaga  ggggacagctttcttgtacaaagtggtcatttaggaaaacctttca |
| --- | --- |
| attB3_3´UTR_dhfr_r  attB4_ech5'UTR_f | ggggacaactttgtataataaagttgcataaaggtatccgcgtggt  GGGGACAACTTTGTATAGAAAAGTTGTTGCTCGCCTTGTTCGAA |
| attB1_ech5'UTR_r | GGGGACTGCTTTTTTGTACAAACTTGTACTGGAAGAAGAAGAAAA |
| attB2_ech3'UTR_f | GGGGACAGCTTTCTTGTACAAAGTGGTTGGGACATTCTTTATTT |
| attB3_ech3'UTR_r | GGGGACAACTTTGTATAATAAAGTTGTTCCTGCCGCCGCGGTAA |
| attB1_Neo_f | GGGGACAAGTTTGTACAAAAAAGCAGGCTATGGGATCGGCCATTGAACA |
| attB2_Neo_r | GGGGACCACTTTGTACAAGAAAGCTGGGTCACACGGCTAGCATACTCTA |
| attB1_Hyg_f | GGGGACAAGTTTGTACAAAAAAGCAGGCTATGAAAAAGCCTGAACTCAC |
| attB2_Hyg_r | GGGGACCACTTTGTACAAGAAAGCTGGGTCAAATTCTGTTCAATGTAAT |
| attB2_1F8Hyg_r | GGGGACCACTTTGTACAAGAAAGCTGGGTACTCTATTCCTTTGCCCTCG |
| attB1_1F8_f | GGGGACAAGTTTGTACAAAAAAGCAGGCTCCGTTGCCAATGTAAACACC |
|  |  |
|  |  |
|  |  |
|  |  |
|  |  |
|  |  |
|  |  |
