# Supplementary material for: Evaluation of high efficiency gene knockout strategies for Trypanosoma cruzi
Source: BMC Microbiol. 2009 May 11;9:90. doi: 10.1186/1471-2180-9-90 (PMC2688506; doi:10.1186/1471-2180-9-90)
Supplement: Additional File 7 — Table S3. Oligonucleotides for one-step-PCR. [file 1471-2180-9-90-S7.doc]

Supplementary table 3. Oligonucleotides for one-step-PCR

| Name | Sequence |
| --- | --- |
| LP_ech_Neo_f | ATGACTCAAAGCTTTGACAGAGTTCCGCACGACACAAAGGGGGAGAGTATAGGGACGCTGCATGTTGTGAACTTTCCCATGGGATCGGCCATTGAACA |
| LP_ech_Neo_r | TTAGTACGGCACGGTCGGATGCCACTGGCGAGCTTGATTCCCCATAGGCTTAAAGTACGCCTCCACATAATCCGCAGTTCAGAAGAACTCGTCAAGAAG |
| LP_ech_Hyg_f | ATGACTCAAAGCCTTGACAGGGTTCCGCACGACACAAAGGGGGAGAGTATAGGAACGCTGCATGTTGTGAACTTTCCCATGAAAAAGCCTGAACTCACC |
| LP_ech_Hyg_r | TTAGTACGGCAAAGTCGGATGCCACTGGCGAGCTTGATTCCCCAAAGGCTTAAAGTACGCCTCCACATAATCCGCAGTTACTCTATTCCTTTGCCCTC |
| LP_dhfr_Neo_f | ATGTCGCTGTTTAAGATCCGCATGCCGGAGACGGTGGCGGAGGGGACACGTCTCGCACTGCGTGCCTTTTCCCTCGTTATGGGATCGGCCATTGAAC |
| LP_dhfr_Neo_r  LP_dhfr-Hyg_f  LP_dhfr-Hyg_r  LP_dhfr-UTR_Neo_f  LP_dhfr-UTR_Neo_r | CTAAACCGCCATCTTCATGGAGATGGGAGGGTATGGCGCATAGTCAATGACCTCCATGTCACCCTCCTCGTAGTCCTCTCAGAAGAACTCGTCAAGAAG  ATGTCGCTGTTTAAGATCCGCATGCCGGAGACGGTGGCGGAGGGGACACGTCTCGCACTGCGTGCCTTTTCCCTCGTTATGAAAAAGCCTGAACTCAC  CTAAACCGCCATCTTCATGGAGATGGGAGGGTATGGCGCATAGTCAATGACCTCCATGTCACCCTCCTCGTAGTCCTCACTCTATTCCTTTGCCCTCG  GAATCCACACAAACAGGCCGGCAAGCGGAAAAGAGGTCGTACGTACTTTTACACTTTTCTCTCACTTGTGCGTCCACCATGGGATCGGCCATTGAAC  CGCCTTTTTACGGTCCCCACGTACTACTTTCGGCTCCCCGTTTCTGGCGCGCACGATTTGAAAGGTTTTCCTAAATGATCAGAAGAACTCGTCAAGAAG |
