# Supplementary material for: Evaluation of high efficiency gene knockout strategies for Trypanosoma cruzi
Source: BMC Microbiol. 2009 May 11;9:90. doi: 10.1186/1471-2180-9-90 (PMC2688506; doi:10.1186/1471-2180-9-90)
Supplement: Additional File 8 — Table S4. Oligonucleotides for probe generation of Southern blot analysis. [file 1471-2180-9-90-S8.doc]

Supplementary table 4. Oligonucleotides for probe generation of Southern blot analysis

| Name | Sequence |
| --- | --- |
| ech1_pb_f | ATGGTGAAAGCCATGCAACG |
| ech1_pb_r | CCCCAAAGGCTTAAAGTACG |
| Hyg_f | ATGAAAAAGCCTGAACTCACC |
| Hyg_r | TACTCTATTCCTTTGCCCTC |
| Neo_f | ATGGGATCGGCCATTGAACA |
| Neo_r | TCAGAAGAACTCGTCAAGAAG |
|  |  |
|  |  |
